# Mesophyll conductance and N allocation co-explained the variation in photosynthesis in two canola genotypes under contrasting nitrogen supply

**DOI:** 10.3389/fpls.2023.1171331

**Published:** 2023-05-08

**Authors:** Jiahuan Liu, Kangkang Zhang, Junguo Bi, Xinqiao Yu, Lijun Luo, Liyong Hu

**Affiliations:** ^1^ Ministry of Agriculture and Rural Affairs (MARA) Key Laboratory of Crop Ecophysiology Farming System in the Middle Reaches of the Yangtze River, College of Plant Science Technology, Huazhong Agricultural University, Wuhan, China; ^2^ Shanghai Agrobiological Gene Center, Shanghai, China

**Keywords:** canola, genotype, photosynthetic nitrogen use efficiency, mesophyll conductance, N allocation, photosynthetic N, nitrogen

## Abstract

The application of nitrogen fertilizer within a normal range has been found to increase the leaf nitrogen content and photosynthetic rate of canola plants (*Brassica napus* L.). Despite numerous studies on the separate effects of CO_2_ diffusion limitation and nitrogen allocation trade-off on photosynthetic rate, few have examined both these factors in relation to the photosynthetic rate of canola. In this study, two genotypes of canola with varying leaf nitrogen content were analyzed to determine the impact of nitrogen supply on leaf photosynthesis, mesophyll conductance, and nitrogen partitioning. The results showed that the CO_2_ assimilation rate (*A*), mesophyll conductance (*g*_m_), and photosynthetic nitrogen content (*N*_psn_) increased with an increase in nitrogen supply in both genotypes. The relationship between nitrogen content and *A* followed a linear-plateau regression, while *A* had linear relationships with both photosynthetic nitrogen content and *g*_m_, indicating that the key to enhancing *A* is increasing the distribution of leaf nitrogen into the photosynthetic apparatus and *g*_m_, rather than just increasing nitrogen content. Under high nitrogen treatment, the genotype (QZ) with high nitrogen content had 50.7% more nitrogen than the other genotype (ZY21), but had similar *A*, which was primarily due to ZY21’s higher photosynthetic nitrogen distribution ratio and stomatal conductance (*g*_sw_). On the other hand, QZ showed a higher *A* than ZY21 under low nitrogen treatment as QZ had higher *N*_psn_ and *g*_m_ compared to ZY21. Our results indicate that, in selecting high PNUE rapeseed varieties, it is important to consider the higher photosynthetic nitrogen distribution ratio and CO_2_ diffusion conductance.

## Introduction

In the past century, the invention of ammonia synthesis technology and its application in agriculture has resulted in an increase in crop yield (Bouchet et al., 2016). However, this advancement has also caused environmental problems such as water eutrophication and soil acidification due to excess nitrogen not being absorbed by crops (Lassaletta et al., 2014). Optimizing agricultural productivity while minimizing environmental impact has become increasingly important, and as a result, improving crop nitrogen use efficiency (NUE) has emerged as a long-term research priority. The definition of NUE is diverse and complex, and in plant ecophysiology, it is generally defined as the CO_2_ assimilation rate per unit leaf N concentration (PNUE). Increasing PNUE is crucial for improving NUE (Garnier et al., 1995).

Leaf CO_2_ assimilation rate (*A*) is closely linked to leaf nitrogen content, as much of the leaf N is involved in the photosynthetic reaction process (Evans, 1989; Terashima et al., 2011; Yamori et al., 2011; Xiong et al., 2015c). Different forms of nitrogen are present in leaves, including free amino acids, nitrates, soluble proteins, cell-wall binding proteins, and membrane proteins. The allocation pattern of leaf nitrogen to each nitrogen component varies among species (Onoda et al., 2017). A trade-off exists between nitrogen allocation to photosynthetic proteins and non-photosynthetic proteins to maintain a high carbon assimilation rate in plant leaves. For instance, deciduous plants have a higher PNUE than evergreen plants as deciduous plants allocate more nitrogen to the photosynthetic system. However, evergreen species allocate more nitrogen into the cell wall to maintain a longer leaf lifespan (Takashima et al., 2004). A study on *Polygonum cuspidatum* Sieb. et Zucc. showed that the smaller allocation of nitrogen to the cell walls in late germinators was associated with a higher CO_2_ assimilation rate, primarily due to a greater fraction of nitrogen allocated to the photosynthetic apparatus (Onoda et al., 2004). The invasive plant, *Ageratina adenophora*, appears to have evolved to allocate a higher fraction of nitrogen to photosynthesis and reduced cell wall allocation (Feng et al., 2009). However, this trade-off is not widely accepted, as other studies in sclerophyllous leaves, between different plant species (Hikosaka and Shigeno, 2009) and among native and invasive species (Funk et al., 2013), did not find this trend. Additionally, the fraction of nitrogen not involved in the metabolic process and structure construction in leaves is referred to as storage nitrogen. It accounts for approximately 50% of the total nitrogen content in plant leaves (Xu et al., 2012; Liu et al., 2018) and plays a crucial role in maintaining leaf expansion and photosynthetic protein synthesis (Lehmeier et al., 2013; Liu et al., 2018). However, a high proportion of storage nitrogen will reduce the PNUE of leaves.

In *C*_3_ plants, the CO_2_ assimilation rate is mainly limited by Rubisco carboxylation capacity and CO_2_ diffusion efficiency (von Caemmerer and Evans, 2010). Rubisco, the most abundant enzyme in plants, is essential for fixing CO_2_ in the Calvin cycle and uses up to 20% of the plant’s nitrogen to maintain high photosynthetic rates in leaves (Evans, 1989; Evans and Clarke, 2019). Under natural conditions, the actual carboxylation reaction of Rubisco is much lower than its potential capacity, particularly in leaves with high nitrogen content, because a large number of Rubisco enzymes are inactive due to a lack of CO_2_ (Cheng and Fuchigami, 2000; Yamori et al., 2011; Li et al., 2013). During the diffusion of CO_2_ from the atmosphere to chloroplasts, the CO_2_ encounters several resistances, and its reciprocal is called CO_2_ diffusion efficiency, including the leaf boundary layer conductance, stomatal conductance to CO_2_ (*g*_sc_), and mesophyll conductance (*g*_m_). Despite many studies showings that increasing nitrogen supply increases *g*_sc_ and *g*_m_ in various *C*_3_ crops (Li et al., 2009; Yamori et al., 2011; Xiong et al., 2015b), the increase in *g*_m_ is much smaller than the increase in Rubisco enzyme content. As a result, the CO_2_ concentration at chloroplast carboxylation sites (*C*_c_) remains insufficient to meet the saturation needs of Rubisco enzymes in high N conditions (Li et al., 2009; Yamori et al., 2011).

There have been numerous studies examining the independent effects of CO_2_ diffusion limitation and N allocation trade-off on *A* and PNUE. However, these two hypotheses are not mutually exclusive and need to be considered together to fully understand their effects on *A* and PNUE (Onoda et al., 2017). Our previous study found that, although two canola varieties (Quanzi & Zheyou 21) showed significant differences in leaf nitrogen content, they had a similar maximum CO_2_ assimilation rate under high light intensity (Liu et al., 2021). Based on this, we hypothesized that the two genotypes might differ in N allocation trade-off and CO_2_ diffusion. To test this hypothesis, we conducted a pot experiment with two canola genotypes under different N supplies. The objective of the study was to (1) Evaluate the impact of different N supplies on *A* and PNUE in the two canola genotypes; (2) Examine the effects of different N supplies on photosynthetic limitation and N partitioning in two canola genotypes; (3) Determine the main cause of the reduction in PNUE under high N supply, whether it is due to CO_2_ diffusion limitation or N allocation trade-off.

## Materials and methods

### Plant materials and N treatments

Two canola genotypes, Quanzi (QZ) and Zheyou 21 (ZY21), were selected. Seeds were provided by the National Key Laboratory of Crop Genetic Improvement and sawed in 0.5 L pots filled with a 0.85 kg mixture of corn soil and sand (1:1, w/w) on the campus of Huazhong Agriculture University, Wuhan, China (114°22’E, 30°29’N). KH_2_PO_4_ was mixed into the mixture at the rate of 275 mg per pot. For high N (HN) and low N (LN) treatments, Urea was applied at 144 mg per pot and 29 mg per pot, respectively. Plants were grown in a growth chamber with 14h day/10h night. The light intensity (photosynthetic photon flux density) was 400-420 µmol m^–2^ s^–1^ at the canopy level, and the temperatures were set to 25/20°C (day/night).

### Gas exchange and chlorophyll fluorescence measurements

After one month of growth, we used an open-flow gas exchange system (Li-Cor 6800; Li‐Cor Inc., Lincoln, NE, USA) with a Multiphase Flash™ Fluorometer (6800-01 A) apertures with 6 cm^2^. To avoid plant rhythm on gas exchange measurements, all plants were measured between 9:00 to 16:00. To minimize leaf position and age effects, measurements were taken on the newest fully expanded leaves. In the leaf chamber, CO_2_ concentration was set at 400 µmol mol^-1^ with a CO_2_ mixer, leaf temperature was maintained at 25 °C, PPFD was 1000 µmol m^–2^ s^–1^ with a blue: red (10:90) light, the leaf-to-air vapor pressure deficit (VPD) was 1.2 kPa. After *A* and stomatal conductance (*g*_sw_) reached the steady-state stage, usually after 20 min, the gas exchange parameters and fluorescence parameters were simultaneously recorded. CO_2_ response curves measurements were performed on three plants for each treatment, and the CO_2_ concentrations in the reference chamber were set across a series of 400, 300, 200, 150, 100, 50, 400, 600, 800, 1000, 1500, 2000 µmol mol^-1^. Then, the maximum carboxylation rate (*V*_c, max_) and maximum electron transport rate (*J*_max_) were calculated using an R package “*plantecophys*” (Duursma, 2015) from the *A*/*C*_c_ response curves according to the FvCB model (Farquhar et al., 1980).

### Estimation of mesophyll conductance

The actual photochemical efficiency of photochemical efficiency of PSII (Φ_PSII_) was calculated as follows:


(1) $${\text{Φ}}_{\text{PSII}}\text{ = }\frac{\left(F_{\text{m}}^{'}\;\text{- }F_{\text{s}}\right)}{F_{\text{m}}^{'}}$$

Where, $F{'}_{m}$ is the maximum fluorescence and *F*_s_ represents the steady-state fluorescence. Then, the electron transport rate (ETR) was calculated as follows:


(2) $${\text{ETR = Φ}}_{\text{PSII}}\times \text{PPFD}\times \alpha \times \beta$$

Where PPFD is photosynthetic photon flux density, *α* is leaf absorbance, *β* represents the distribution ratio of electrons in PSII. The *αβ* was estimated from light response curves in a low O_2_ environment (O_2_<2%). Specifically, under low O_2_ supply conditions, simultaneous measurement of the response of leaf gas exchange, and chlorophyll fluorescence to PPFD. During the measurements, the leaf chamber conditions were the same as those described above, except that the PPFD was set across a series of 1500, 1000, 800, 400, 250, 150, 100, 50, 20, and 0 µmol m^–2^ s^–1^. Then, the slope of the relationship between Φ_PSII_ and Φ_CO2_ is regarded as the value of *αβ* (Valentini et al., 1995).

The variable *J* method described by Harley et al. (1992) was used to calculate CO_2_ concentration in the chloroplast (*C*_c_) and then to estimate *g*_m_. The *g*_m_ was calculated as follows:


(3) $$C_{c}\text{ = }\frac{{\text{Γ}}^{*}\;\left(ETR + 8\left(\text{A + }R_{\text{d}}\right)\right)}{ETR - 4\left(\text{A + }R_{\text{d}}\right)}$$

(4) $$g_{\text{m}}=\frac{A}{C_{\text{i}}\text{ - }C_{\text{c}}}$$

(5) $${\text{Γ}}^{*}{\text{ =C}}_{\text{i}}^{*}\text{ +}\frac{R_{\text{d}}}{g_{\text{m}}}$$

Where *A*, *C*_i,_ and ETR were determined as previously described, and day respiration rate (*R*_d_) and the CO_2_ compensation point in the absence of mitochondrial respiration (Γ^*^) were calculated by the Laisk method (Harley et al., 1992). Briefly, the response of *A* to *C*_i_ was performed, with CO_2_ concentrations in the reference chamber set across a series of 110, 90, 70, and 50 µmol mol^-1^, at three PPFD of 250, 100, 50 µmol m^–2^ s^–1^, respectively. The intersection point of three *A*/*C*_i_ curves on the *x-*axis was *C*_i_^*^, and on the y-axis was *R*_d_. There was no difference in *R*_d_ values between treatments leaves, thus the average value (0.78 µmol m^–2^ s^–1^) of all the treatments were used in the current study.

### Photosynthetic limitation analysis

Relative limitations of photosynthesis, including stomatal (*l*_s_), mesophyll (*l*_m_), and biochemical (*l*_b_) relative limitations, were calculated as follows Grassi and Magnani (2005):


(6) $$l_{\text{s}}=\frac{g_{\text{t}}/g_{\text{sc}}\cdot \partial \text{A/}\partial {\text{C}}_{\text{c}}}{g_{\text{t}}\text{ + }\partial \text{A/}\partial {\text{C}}_{\text{c}}}$$

(7) $$l_{m}=\frac{{\text{g}}_{\text{t}}{\text{/ g}}_{\text{m}}\cdot \partial \text{A/}\partial {\text{C}}_{\text{c}}}{{\text{g}}_{\text{t}}\text{ + }\partial \text{A/}\partial {\text{C}}_{\text{c}}}$$

(8) $$l_{b}=\frac{g_{\text{t}}}{g_{\text{t}}\text{ + }\partial \text{A/}\partial {\text{C}}_{\text{c}}}$$

Where *g*_t_ is the total conductance to CO_2_ (*g*_t_ = 1/(1/*g*_sc_ + 1/*g*_m_)), *g*_sc_ is stomatal conductance to CO_2_ (*g*_sc_ = *g*_sw_/1.6), and *A*/*C*_c_ is the slope of *A* versus *C*_c_ response curve. In the current study, *A*/*C*_c_ was calculated according to the FvCB model (Farquhar et al., 1980):


(9) $$\partial \text{A/}\partial {\text{C}}_{\text{c}}\text{ =}V_{\text{c, max}}\frac{{\text{Γ}}^{*}\text{ + }K_{\text{c}}\text{(1 + O/}K_{\text{o}})}{{{\text{(C}}_{\text{c}}\text{ + }K_{\text{c}}\text{(1 + O/}K_{\text{o}}\text{))}}^{2}}$$

Where *K*_c_ and *K*_o_ are the Rubisco Michaelis-Menten constants for CO_2_ and O_2_, respectively.

### N partitioning by function

The leaf N is divided into photosynthetic N, respiration N, structural N, and storage nitrogen based on the LUNA model described by (Xu et al., 2012; Ali et al., 2016). Photosynthetic N is further divided into three major classes of proteins, including carboxylation system protein (*N*_cb_), electron transport system protein (*N*_et_), and light capture system protein (*N*_lc_). Respiration N is the respiratory enzyme located in the mitochondria to generate the energy required for plant growth. Structural N (*N*_str_) represents the N used to build cell walls and DNA. In the current study, structural N was measured by SDS-insoluble protein N. Leaf structural N was measured according to the method of (Takashima et al., 2004) with minor modifications. Leaf tissue was harvested about 0.1g and immersed in liquid N and stored in an ultracold storage freezer (-80). The frozen leaf samples were ground with a Mixer Mill (MM 400, Retsch, Haan, Germany) and homogenized with 1 ml of 100 mM Na phosphate buffer [PH 7.4 and containing 0.4M D-sorbitol, 2 mM MgCl_2_, 10 mM NaCl, 5 mM iodoacetate, 5 mM phenylmethylsulphonyl fluoride, and 5 mM DTT], then washed into a centrifuge tube with 4 ml of phosphate buffer. The samples were centrifuged at 15 000 g, 4 for 15 min; after that, 1 ml of phosphate buffer containing 3% sodium dodecyl sulfate (SDS) was added to the residue, followed by heating at 90 for 5 min. And then, the mixture was separated by centrifugation at 4500 g for 10 min. This procedure was repeated four times, and the residue (regarded as SDS-insoluble protein) was washed to the quantitative filter paper with ethanol. The samples on the filter papers were dried naturally in a fume hood. And then, the structural N on the quantitative filter paper was dried and digested with H_2_SO_4_-HClO_4_ according to the method of Gallaher et al. (1976). The N concentration in the digestion solution was measured by a discrete analyzer (SmartChem 200, Unity Scientific, Brookfield, CT). The blank quantitative filter paper was used as blank control. Besides, about 0.1 g dry matter was used to determine the total leaf N concentration with the same method described above. Storage N is equal to the total leaf N minus photosynthetic N, respiration N, and structural N. The calculation formulas for each form of N are given below.

The carboxylation N content (*N*_cb_) is:


(10) $$N_{\text{cb}}=\frac{V_{\text{c, max}}}{6.25 \times V_{\text{cr}}}$$

Where 6.25 [g Rubisco (g^-1^ N)] represents the proportion of N allocated to Rubisco, *V*_cr_ is the maximum rate of RUBP carboxylation per unit Rubisco protein [20.8 µmol CO_2_ (g Rubisco)^-1^s^-1^ at 25] (Niinemets and Tenhunen, 1997).

The electron transport N (*N*_et_) content is:


(11) $$N_{\text{et}}\text{ =}\frac{J_{\text{max}}}{8.06 \times J_{\text{mc}}}$$

*J*_mc_ is the maximum electron transport rate per unit cytochrome *f* [155.65 µmol e^-1^ µmol cytochrome *f* s^-1^ at 25].

The light capture N (*N*_lc_) content:


(12) $$N_{\text{lc}}\text{ = }\frac{C_{\text{Chl}}}{C_{\text{B}}}$$

*C*_Chl_ is the leaf chlorophyll concentration (mmol m^-2^), and *C*_B_ is the ratio of chlorophyll to organic N in light-harvesting components (2.15 mmol g^-1^).

The photosynthetic N content (*N*_psn_) is:


(13) $$N_{\text{psn}}\text{ = }N_{\text{cb}}\text{ + }N_{\text{et}}\text{ + }N_{\text{lc}}$$

The respiratory N content (*N*_resp_) is:


(14) $$N_{\text{resp}}\text{ = }\frac{R_{\text{n}}}{33.69}$$

*R*_n_ is the leaf dark respiration rate (µmol CO_2_ m^-2^ s^-1^), which was assumed to be twice that of *R*_d_ (Yamori et al., 2005), and 33.69 is the leaf N use efficiency for respiration at 25 (µmol CO_2_ g^-1^ N s^-1^).

The storage N content (*N*_store_) is:


(15) $$N_{\text{store}}\text{ = }N_{\text{a}}\;\text{- }N_{\text{psn}}\;\text{- }N_{\text{resp}}\;\text{- }N_{\text{str}}$$

*N*_a_ is the leaf total N concentration based on the area, *N*_str_ is the leaf structural N content, which is also the SDS-insoluble N content in the current study (Takashima et al., 2004).

### Data analysis

Two-way ANOVA was used on two canola varieties to test the trait response to nitrogen treatments. Linear-plateau regression and linear regression were performed to test the correlations between trait parameters. All analyses and figures were performed in R version 4.2.2 (R Core Team, 2020).

## Results

### Leaf morphological and physiological traits

The results of our study indicated that the application of high levels of nitrogen significantly enhanced the growth parameters of both QZ and ZY21 varieties. The dry matter weight (DMW), shoot weight (SW), and root weight (RW) of both varieties increased by 120.5% and 184.5%, 141.3% and 194.6%, and 92% and 168.7%, respectively, under high N treatment compared to low N treatment (**Table 1**). The results showed that ZY21 had a higher response to high N treatment, with 65.6%, 72.5%, and 54.9% higher DMW, SW, and RW, respectively, compared to QZ. The leaf nitrogen concentration (*N*_a_) of plants under high N treatment was found to be 1.5-fold and 1.6-fold higher in QZ and ZY21, respectively, compared to plants under low N treatment. Under low N treatment, the difference in *N*_a_ between the two varieties was not statistically significant, but under high N treatment, the *N*_a_ of QZ was found to be significantly higher than that of ZY21 (by 50.7%). Our results also demonstrate that high N treatment increased the SPAD value and chlorophyll content based on leaf area (*C*_Chl_) in both QZ and ZY21, with an increase of 18.3% and 22.0% in SPAD value, and 80.3% and 78.4% in *C*_Chl_, respectively (**Table 2**).

**Table 1:** Effects of N supply on canola (Brassica napus L.) growth.

Nitrogen	Variety	DMW	SW	RW
		g	g	g
HN	QZ	3.66 ± 1.56	2.22 ± 1.30	1.44 ± 0.38
	ZY21	6.06 ± 1.49	3.83 ± 1.04	2.23 ± 0.66
LN	QZ	1.66 ± 0.42	0.92 ± 0.21	0.75 ± 0.30
	ZY21	2.13 ± 0.69	1.3 ± 0.40	0.83 ± 0.34
ANOVA				
Nitrogen (N)		**	**	**
Variety (V)		*	*	*
N * V		ns	ns	ns

HN, high nitrogen treatment; LN, low nitrogen treatment; DMW, dry matter weight; SW, shoot weight; RW, root weight. Data are presented as mean ± SD (n = 3). Stars denote a significant change in values (ns, no significant; *P< 0.05; **P< 0.01).

**Table 2:** Leaf SPAD value, chlorophyll content (*C*_Chl_), and area-based leaf N content *N*_a_) of *Brassica napus* L. under different N supply.

Nitrogen	Variety	SPAD	*C*_Chl._	*N_a_*
			(mg m^-2^)	(g m^-2^)
HN	QZ	65.2 ± 1.1	858 ± 110	3.03 ± 0.63
	ZY21	56.6 ± 0.7	591 ± 46	2.01 ± 0.10
LN	QZ	55.1 ± 1.6	476 ± 21	1.49 ± 0.07
	ZY21	46.4 ± 4.4	331 ± 14	1.25 ± 0.23
ANOVA				
Nitrogen (N)		**	**	**
Variety (V)		**	**	*
N * V		ns	ns	ns

Note. HN, high nitrogen treatment; LN, low nitrogen treatment. Data are presented as mean ± SD (n = 3). Stars denote a significant change in values (ns, no significant; *P< 0.05; **P< 0.01).

### Effect of N concentration on photosynthesis and CO_2_ diffusion efficiency

**Figure 1:**
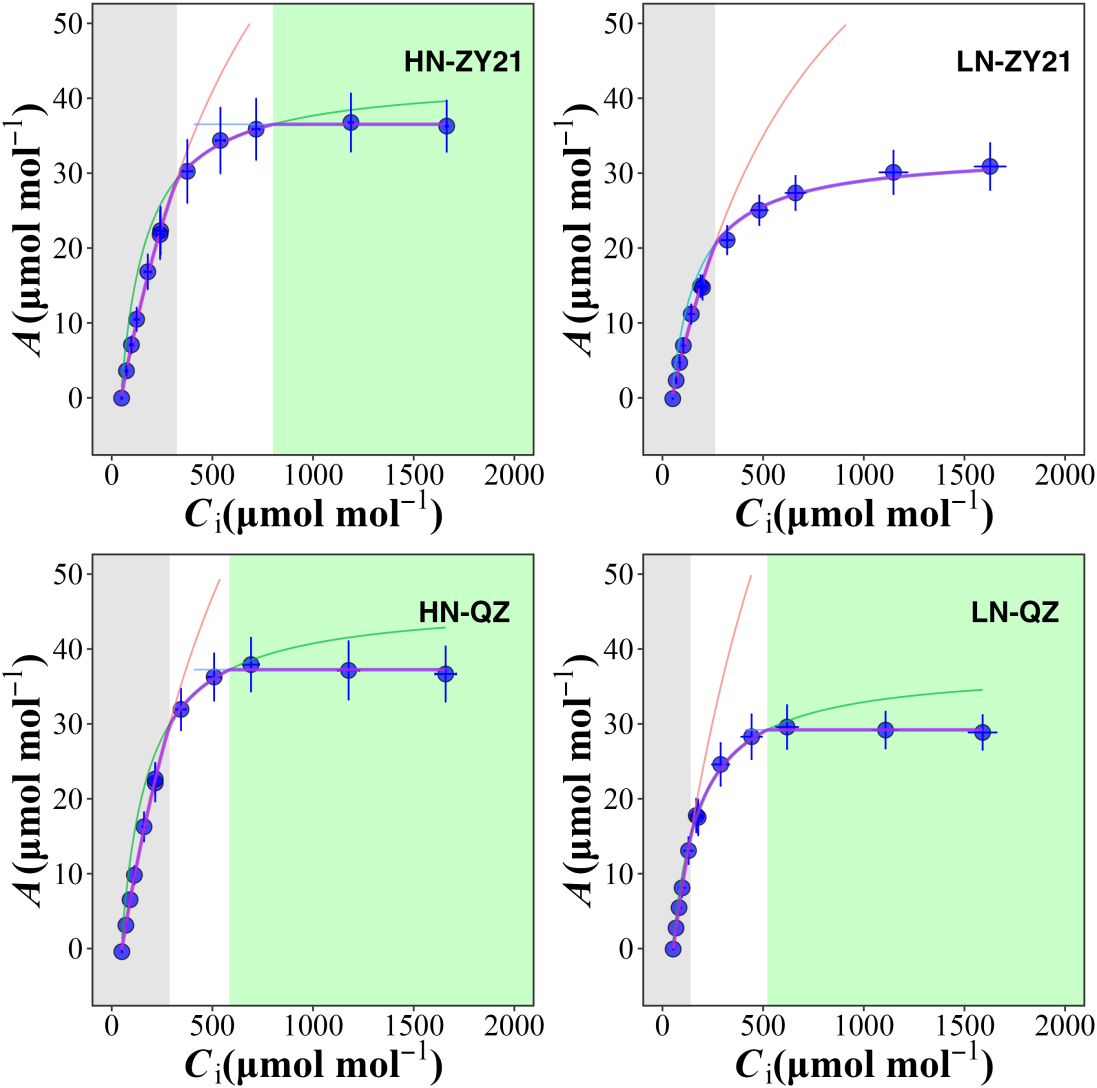
Chloroplast CO_2_ concentration (*C*_c_) response of the net photosynthetic rate (A) for *Brassica napus* L. under different nitrogen supplies. *A* is limited by RuBP carboxylation (grey shaded area), RuBP regeneration (white area), and triose-phosphate utilization (green area). RuBP carboxylation rate (red lines), RuBP regeneration rate (green lines), and triose-phosphate utilization rate (blue lines) were estimated using FvCB leaf photosynthesis model. The red dots represent the net photosynthetic rate when the ambient CO_2_ concentration is 400 μmol mol^-1^.

The results of our study indicated that the supply of nitrogen has a significant impact on CO_2_ response curves (**Figure 1**) and photosynthetic parameters (**Table 3**). Compared with high N treatment, the CO_2_ assimilation rate (*A*), mesophyll conductance of CO_2_ (*g*_m_), maximum carboxylation rate (*V*_c, max_), and maximum electron transport rate (*J*_max_) of the two varieties were significantly different in response to low N treatment. Specifically, as compared to plants supplied with high N level, *A*, *V*_c, max,_ and *J*_max_ of QZ supplied with low N level decreased by 18.0%, 36.2%, and 30.6% whereas, those of ZY21 supplied with low N level decreased by 37.7%, 35.5%, and 27.6%. Besides, the *g*_m_ of QZ is hardly affected by nitrogen fertilizer supply, but that of ZY21 is significantly reduced under low nitrogen conditions. This result indicated that the photosynthetic parameters of ZY21 were more sensitive to the change of nitrogen supply than that of QZ. High nitrogen fertilizer supply decreased the *C*_c_ at process transition between Rubisco carboxylation-limited (*W*_c_) and RuBP regeneration-limited (*W*_j_) photosynthetic rate. Compared with ZY21, QZ had lower *C*c at the process transition between *W*_c_ to *W*_j_, especially under a low nitrogen fertilizer supply. Furthermore, increasing the N supply significantly reduced the photosynthetic nitrogen use efficiency (PNUE) of QZ by 39.1%, whereas the PNUE of ZY21 was less affected by N supplies (**Table 3**). Compared with QZ, ZY21 had a higher PNUE under high N treatment; the PNUE of ZY21 is 53.0% higher than QZ.

**Table 3:** Effects of different N supplies on the CO_2_ assimilation rate(A), stomatal conductance (g_sw_), mesophyll conductance of CO_2_ (g_m_), intercellular CO_2_ concentration (C_i_), CO_2_ concentration in the chloroplast (C_C_), maximum carboxylation rate (V_c, max_), maximum electron transport rate (J_max_) and photosynthetic N use efficiency (PNUE) of *Brassica napus* L.

Nitrogen	Variety	*A*	> *g*_sw_	*g*_m_	> *C*_i_	> *C*_c_	> *V*_c, max_	> *J*_max_	PNUE
		(μmol m^-2^ s^-1^)	(mol m^-2^ s^-1^)	(mol m^-2^ s^-1^)	(μmol mol^-1^)	(μmol mol^-1^)	(μmol m^-2^ s^-1^)	(μmol m^-2^ s^-1^)	(μmol g^-1^)
HN	QZ	21.7 ± 2.3	0.320 ± 0.072	0.261 ± 0.026	253 ± 14	149 ± 14	138.9 ± 6.0	233.9 ± 22.8	7.3 ± 0.9
	ZY21	22.3 ± 3.3	0.512 ± 0.052	0.464 ± 0.075	292 ± 8	243 ± 21	98.7 ± 17.4	200.2 ± 31.6	11.1 ± 1.2
LN	QZ	17.8 ± 2.2	0.367 ± 0.153	0.288 ± 0.096	285 ± 19	219 ± 33	88.6 ± 3.8	162.1 ± 13.6	11.9 ± 1.0
	ZY21	13.9 ± 0.5	0.348 ± 0.032	0.234 ± 0.039	309 ± 7	248 ± 16	63.6 ± 2.6	148.6 ± 18.1	11.4 ± 2.1
ANOVA									
Nitrogen (N)		**	ns	*	ns	ns	**	**	*
Variety (V)		ns	ns	ns	ns	**	**	ns	ns
N * V		ns	ns	**	ns	ns	ns	ns	*

Note. HN, high nitrogen treatment; LN, low nitrogen treatment. Data are presented as mean ± SD (n = 3). Stars denote a significant change in values (ns, no significant; *P< 0.05; **P< 0.01).

The quantitative limitation analysis showed that, on average, *A* was mainly limited by biochemical factors (*l*_b_, 60.9%), followed by stomatal conductance (*l*_s_, 21.9%), and mesophyll conductance limitation (*l*_m_, 17.2%) was the lowest (**Figure S1**). The results also suggested that the *A* of QZ is primarily limited by CO_2_ diffusion efficiency, whereas ZY21 is predominantly constrained by biochemical factors.

### Effect of N concentration on leaf N partitioning by function

**Figure 2:**
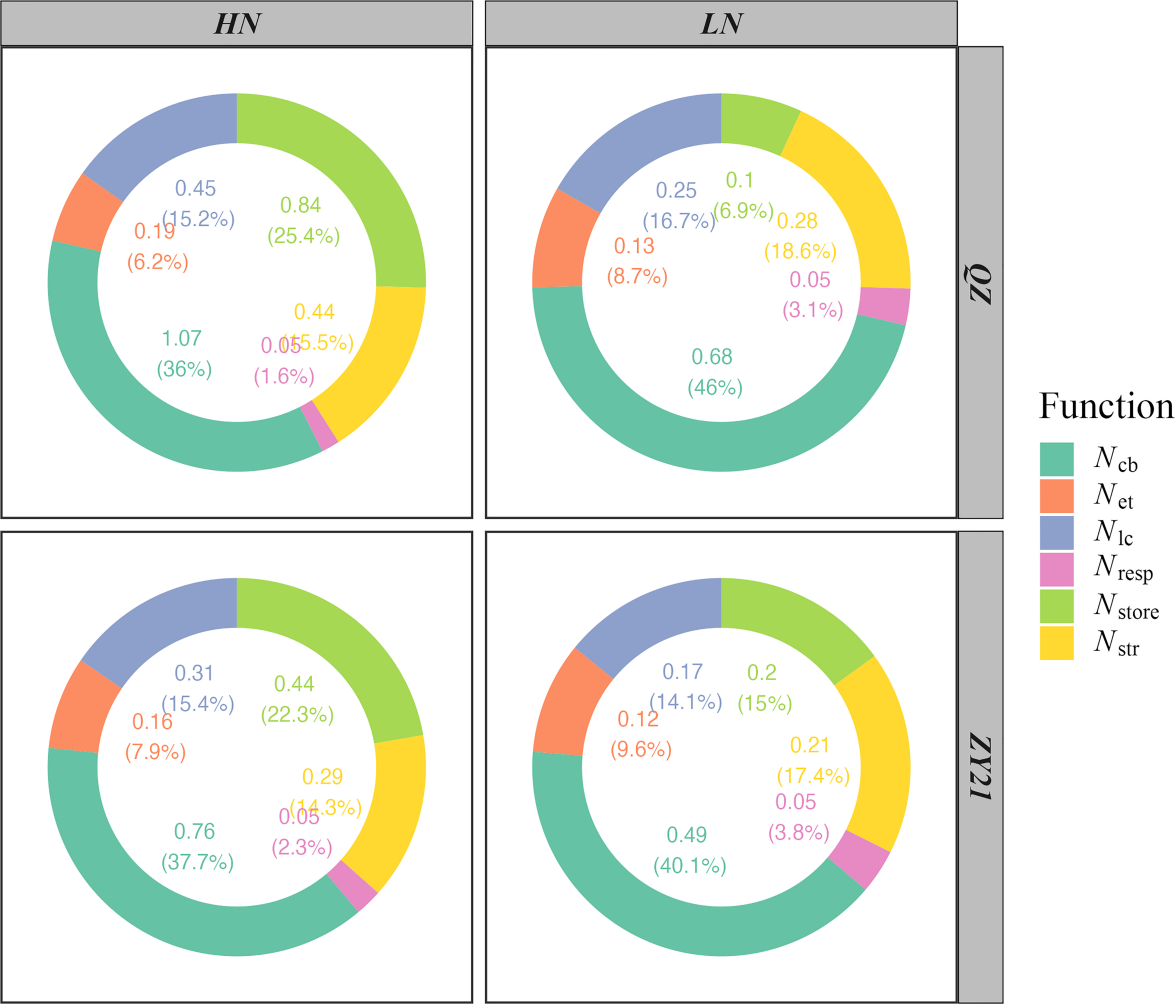
Effects of N supply on N partitioning by the function of two canola (*Brassica napus* L.) varieties (QZ and ZY21).

The results of our study demonstrated the impact of increasing nitrogen supply on the allocation of nitrogen in both QZ and ZY21 varieties. The photosynthetic N (*N*_psn_), respiratory N (*N*_resp_), structural N (*N*_str_), and storage N (*N*_store_) for leaves developed by plants grown under HN were higher than those for plants grown under LN (**Figure 2**). QZ allocated more nitrogen to storage N than ZY21 under high N treatment, while the opposite was observed under low N conditions. The allocation of *N*_psn_ into three major classes of proteins, including carboxylation system protein (*N*_cb_), electron transport system protein (*N*_et_), and light capture system protein (*N*_lc_), was also analyzed. The results showed that with the increase of nitrogen rate, the absolute *N*_cb_, *N*_et_, and *N*_lc_ increased in both varieties (**Figure 2**). However, the relative content of *N*_cb_, *N*_et_, and *N*_lc_ decreased with the rise of nitrogen rate, except for the *N*_lc_ of ZY21. In addition, our results showed that the decrease in the relative content of *N*_cb_ was greater in QZ compared to ZY21 with the increase of nitrogen application (from 46% to 36% in QZ compared to 40.1% to 37.7% in ZY21).

### Correlations between leaf traits

**Figure 3:**
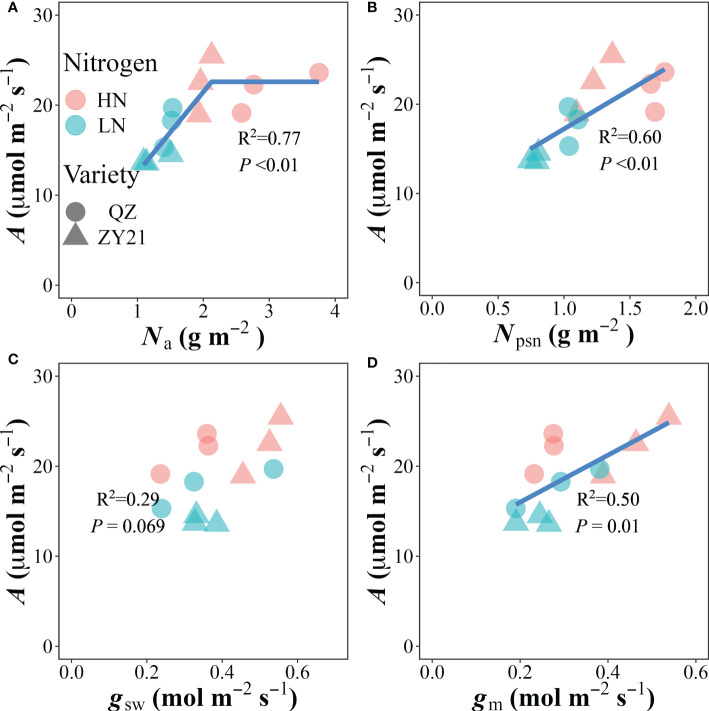
Correlations of **(A)** leaf nitrogen content (*N*_a_), **(B)** photosynthetic nitrogen content (*N*_psn_), **(C)** stomatal conductance (*g*_sw_) and **(D)** mesophyll conductance (*g*_m_) with the CO_2_ assimilation rate (*A*). The data from all treatments are used together for correlation analysis and fitted by linear-plateau **(A)** and linear regression, respectively.

The relationship between photosynthetic rate (*A*) and the concentration of nitrogen based on leaf area (*N*_a_) was found to be positive at *N*_a_ values below 2.12 g m^-2^, and stabilized at the highest value of 22.6 μmol m^-2^ s^-1^ when *N*_a_ exceeded that level (**Figure 3A**). Our analysis also revealed that the photosynthetic N (*N*_psn_), stomatal conductance (*g*_sw_), and mesophyll conductance (*g*_m_) were significantly correlated with *A* in a linear manner (**Figures 3B–D**). The photochemical N utilization efficiency (PNUE) was primarily influenced by the allocation of N in the plant. The results showed that the relative content of N allocated to the carboxylation system protein (*N*_cb%_), electron transport system protein (*N*_et%_), photosynthetic N (*N*_psn%_), and respiratory N (*N*_resp%_) were positively correlated with PNUE. In contrast, the relative content of N allocated to storage (*N*_store%_) was negatively correlated with PNUE. These findings suggested that a higher allocation of N to the photosynthetic and respiratory systems is beneficial for improving PNUE (**Figure S2**).

## Discussion

### N supplement enhanced canola photosynthetic capacity and growth

In the present study, *A* was improved by more N fertilizer supply, which results in larger biomass in both canola genotypes (**Table 1**). Previous studies have observed a strong correlation between *N*_a_ and *A* across a wide range of species (Cheng and Fuchigami, 2000; Yamori et al., 2011; Xiong et al., 2015c) and under different N supplements (Li et al., 2009; Xiong et al., 2015c; Hou et al., 2019). We also found that increasing N fertilizer application significantly increased the *A* in both canola genotypes, but the difference in *N*_a_ between the two genotypes under high nitrogen supply did not cause changes in *A*. Our results suggested that *N*_a_ is not the main limiting factor for *A* under normal fertilizer supply conditions, but canola genotypes with high *N*_a_ have a higher *A* under a low N supply.

### Effects of N partitioning trade-off on A and PNUE

The distribution of nitrogen within the cellular organelles of a *C*_3_ plant can vary significantly. In mature leaves, the average distribution is 75% in chloroplasts, 10% in cell walls, and the remaining 25% is mainly found in mitochondria, peroxisome, and cytosol (Evans and Clarke, 2019). Several previous studies have shown a strong correlation between the allocation ratio of N in the photosynthetic apparatus and *A* (Onoda et al., 2004; Takashima et al., 2004; Hou et al., 2019). Our current study supports this correlation (**Figure 3B**) and shows that the partitioning ratio of nitrogen into the photosynthetic apparatus increased under a low nitrogen supply. As described in the material and method, photosynthetic N is divided into three fractions based on their function, namely: *N*_cb_, *N*_et_, and *N*_lc_. Compared with the low N treatment, the leaf N partitioning ratio into the three components was decreased under high N supply (**Figure 2**), in general, which was recognized as the primary reason for the low PNUE of plants under high N supply (Takashima et al., 2004; Li et al., 2009; Onoda et al., 2017). The results of our study showed that ZY21 demonstrates a comparable net photosynthetic rate due to its efficient distribution of *N*_a_ into *N*_cb_, despite having lower *N*_a_ than QZ. Consequently, ZY21 had a higher PNUE than QZ under high N treatment. These findings highlight the importance of the distribution pattern of *N*_a_ within the photosynthetic apparatus on PNUE in plants (Poorter and Evans, 1998; Takashima et al., 2004; Liu et al., 2018; Hou et al., 2019).

Structural N is thought to make up a significant portion of leaf N, with estimates ranging from 2% to 20% across different species (Takashima et al., 2004; Feng et al., 2008; Harrison et al., 2009). Previous studies on canola have reported that the proportion of N in cell walls ranges from 3% (Hou et al., 2019) to 8% (Liu et al., 2018). The current study found that, on average for all N treatments of both canola genotypes, 16% of leaf N was allocated to structure, which is higher than previous studies and highlights the significant intraspecific variation in structural N. A trade-off between N allocation to cell walls and photosynthetic apparatus has been reported in deciduous herb species such as *Polygonum cuspidatum* (Onoda et al., 2004), *Ageratina adenophora* (Feng et al., 2009), and *Pinus massoniana* (Guan and Wen, 2011). However, not all studies support this trade-off, as some (Harrison et al., 2009; Hikosaka and Shigeno, 2009; Funk et al., 2013) including the current study did not observe such a trend.

Storage N is not involved in any biochemical processes and serves mainly as a buffer pool to support leaf growth and synthesize photosynthetic enzymes (Lehmeier et al., 2013; Liu et al., 2018). In canola, storage N has been found to constitute 30-45% of leaf N (Liu et al., 2018; Hou et al., 2019), while this value was 17.4% on average for both canola genotypes in the current study. Another reason for the numerical differences, in addition to the difference in *N*_str_ measurement values, is that neither study accounted for the potential impact of *g*_m_ on *V*_c, max,_ and *J*_max_ during the fitting of the *A*/*C*_c_ curves, resulting in an underestimation of both values (Sun et al., 2014). The relative content of *N*_psn_ was negatively correlated with the relative content of *N*_store_ in both genotypes under two N supplies (**Figure S2**), indicating a trade-off between *N*_store_ and *N*_psn_. Under N deficiency, new leaves require more N for growth and expansion, leading to a decrease in *N*_store_ (Liu et al., 2018). There, an adequate amount of *N*_store_ can delay leaf senescence and promote leaf expansion, but a higher proportion of *N*_store_ can be disadvantageous for leaf photosynthesis when soil nitrogen is limited.

### Effects of CO_2_ diffusion resistance on A and PNUE

The CO_2_ diffusion pathway is composed of three components: boundary layer conductance, stomatal conductance (*g*_s_), and mesophyll conductance (*g*_m_). The boundary layer has very little resistance to CO_2_ diffusion when plants grow without stress, its limitation on *A* is often ignored in the studies. Our result showed that nitrogen deficiency had little effect on *g*_s_ (**Table 3**), which is in line with previous studies on canola (Hu et al., 2019), rice (Li et al., 2009), and wheat (Veres et al., 2017). However, the generalization of this effect is not yet apparent, as some studies have found that increasing N supply increases *g*_s_ in rice (Xiong et al., 2015b), wheat (Zhang et al., 2017), and oak (Zhu et al., 2020). This discrepancy may be due to differences in the response of *g*_s_ to nitrogen starvation between species or varieties. The duration of nitrogen starvation and the plant size during nitrogen starvation treatment maybe also affect the relationship between nitrogen fertilizer and *g*_sw_.

Leaf anatomy is believed to play a crucial role in determining mesophyll conductance (*g*_m_). Generally, decreasing cell wall thickness (*T*_cw_) or increasing the chloroplast surface area facing the intercellular airspace per unit leaf area (*S*_c_) has been shown to increase *g*_m_ (Evans et al., 2009; Terashima et al., 2011; Muir et al., 2014). Previous studies have demonstrated that increasing leaf nitrogen content can decrease *T*_cw_ and increase *S*_c_, thereby increasing the efficiency of CO_2_ transport across the membrane (Li et al., 2009; Li et al., 2013). We can infer that cell wall thickness is positively correlated with structural protein N, as various structural proteins are closely associated with the cell wall. In both genotypes studied, leaves under low N treatment allocated more N to structural protein N compared to high N treatment, leading to thickening of the cell walls (**Figure 2**). This increase in *T*_cw_ is a likely reason for the decrease in *g*_m_ under N deficiency. Generally, *S*_c_ is related to the chloroplast size and number. Although we did not study chloroplast structure, our results showed that chlorophyll content decreased significantly under N starvation conditions, which is closely related to the number of chloroplasts (Xiong et al., 2015a). These changes in *S*_c_ also explain the decrease in *g*_m_ under low N supply. Besides, in the present study, the response of the photosynthetic nitrogen use efficiency (PNUE) of two canola genotypes to nitrogen fertilizer treatment was different. The N supplement significantly decreased the PNUE of QZ, but had little effect on ZY21 (**Table 3**). This result is probably due to the increase in *g*_m_ of QZ under high N treatment being lower than the increase in Rubisco enzyme content of leaves, which resulted in a lower CO_2_ concentration in the chloroplast (*C*_c_) (**Table 3**). In comparison, the high N supply resulted in a higher *C*_c_ in ZY21 than low N treatment (**Table 3**), which offsets the increase in Rubisco enzyme content.

## Conclusion

The present study demonstrated that the decrease in the allocation ratio of N into photosynthetic apparatus and the insufficient supply of CO_2_ at chloroplast carboxylation sites are the main reasons leading to the decline in PNUE under high N supply in both genotypes. Besides, under high N application, the canola genotype with higher N content (QZ) allocated more N to the storage N and showed lower PNUE, which is not conducive to high-yield cultivation and can not meet the production demand. Hence, optimizing the N partitioning and enhancing *g*_m_ in plant leaves is expected to increase canola’s net photosynthetic rate. These findings could have practical implications in developing strategies for enhancing canola productivity and sustainability.

## Data availability statement

The original contributions presented in the study are included in the article/**Supplementary Material**. Further inquiries can be directed to the corresponding authors.

## Author contributions

JL and LH planned and designed the research. JL and KZ performed the experiments. JL analysed the data. JL, KZ, JB, XY, LL, and LH wrote and revised the manuscript. All authors contributed to the article and approved the submitted version.
