# Supplementary material for: Mesophyll conductance and N allocation co-explained the variation in photosynthesis in two canola genotypes under contrasting nitrogen supply
Source: Front Plant Sci. 2023 May 8;14:1171331. doi: 10.3389/fpls.2023.1171331 (PMC10202220; doi:10.3389/fpls.2023.1171331)
Supplement: Supplementary file 1 [file Image_1.pdf]

# *Supplementary Material*

**Mesophyll conductance and N allocation co-explained the variation in photosynthesis in two canola genotypes under contrasting nitrogen supply**

Jiahuan Liu [†, 1], Kangkang Zhang [†, 2], Junguo Bi [2], Xinqiao Yu[2], Lijun Luo [*, 2] and Liyong Hu [*, 1]

[1] MARA Key Laboratory of Crop Ecophysiology and Farming System in the Middle Reaches of the Yangtze River, College of Plant Science and Technology, Huazhong Agricultural University, Wuhan 430070, China

[2] Shanghai Agrobiological Gene Center, No. 2901 Beidi Road, Shanghai 201106, China

[†] These authors contributed equally to this work and share first authorship

[*] **Correspondence**

Liyong Hu

liyonghu@mail.hzau.edu.cn

Lijun Luo

lijun@sagc.org.cn

## Supplementary Figures

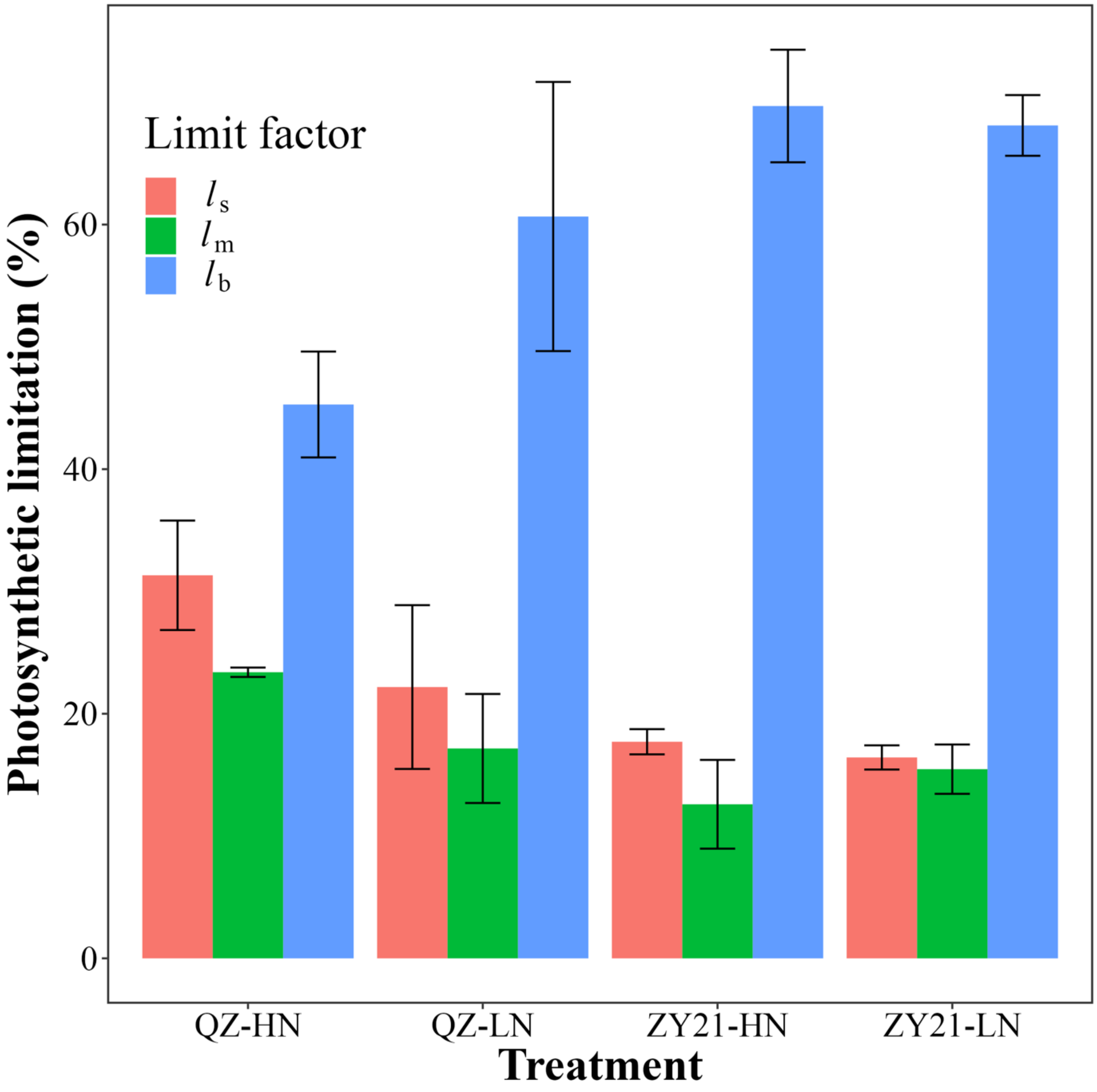


**Fig. S1.** Quantitative limitation analysis of photosynthetic $CO_2$ assimilation of QZ and ZY21 under two N treatments. The total relative photosynthetic limitation was composed of stomatal conductance ($l_s$), mesophyll conductance ($l_m$), and biochemical ($l_b$) limitations. Bars represent the means ± SD (n = 3).

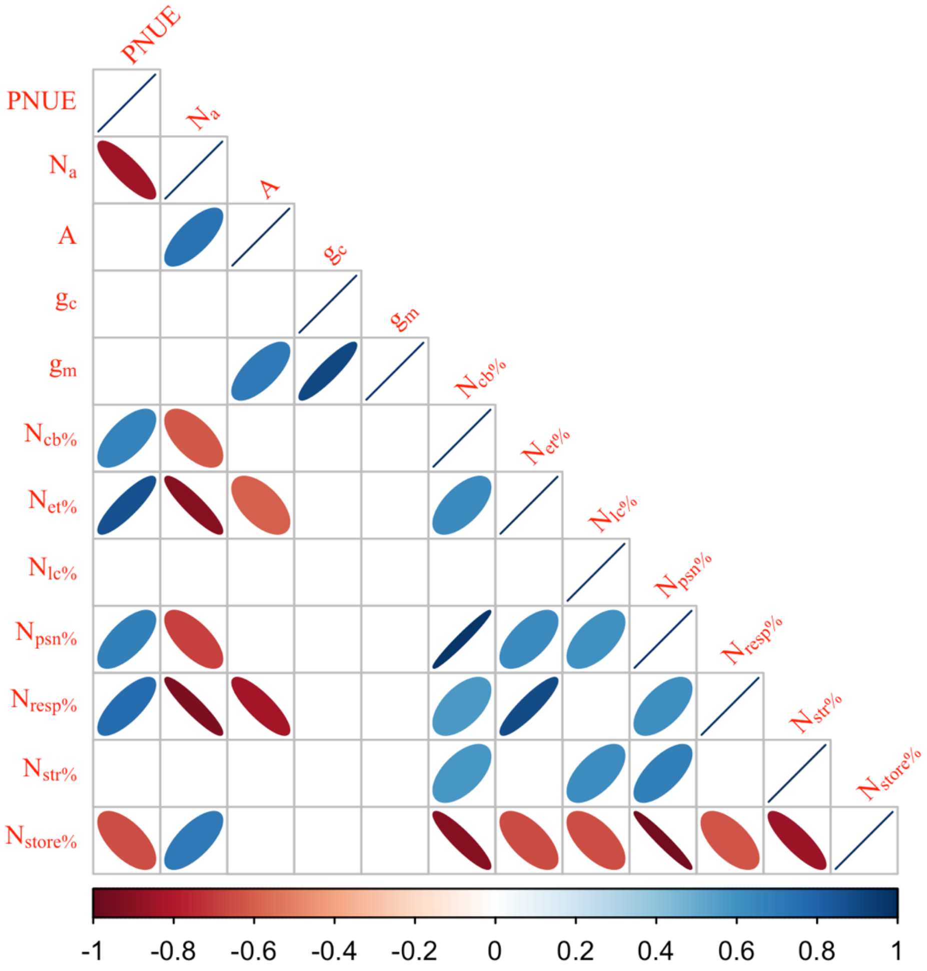


**Fig. S2.** Correlation matrix between N allocation, photosynthesis, and photosynthetic nitrogen use efficiency (PNUE). $N_a$, nitrogen concentration based on the area; $A$, net photosynthetic rate; $g_c$, stomatal conductance to $CO_2$; $g_m$, mesophyll conductance to $CO_2$; $N_{cb\%}$, relative carboxylation system protein N; $N_{et\%}$, relative electron transport system protein N; $N_{lc\%}$, relative light capture system protein; $N_{psn\%}$, photosynthetic N; $N_{resp\%}$, relative respiratory N; $N_{store\%}$, relative storage N; $N_{str\%}$, relative structural N. The data from all N treatments are used together for correlation analysis. The linear model estimated the correlations, and the ellipses were drawn under the 95% confidence level.
